# Tob1 induces apoptosis and inhibits proliferation, migration and invasion of gastric cancer cells by activating Smad4 and inhibiting β-catenin signaling

**DOI:** 10.3892/ijo.2012.1517

**Published:** 2012-06-12

**Authors:** JUTHIKA KUNDU, S.M. RIAJUL WAHAB, JOYDEB KUMAR KUNDU, YOON-LA CHOI, OZGUR CEM ERKIN, HUN SEOK LEE, SANG GYU PARK, YOUNG KEE SHIN

**Affiliations:** 1 Research Institute of Pharmaceutical Sciences, College of Pharmacy, Seoul National University, Seoul;; 2 College of Pharmacy, Keimyung University, Daegu;; 3 Department of Pathology, Samsung Medical Center, Sungkyunkwan University School of Medicine, Seoul;; 4 Department of Biomedical Science, College of Life Science, CHA University, Sungnam-si, Gyunggi-do;; 5 Advanced Institute of Convergence Technology (AICT), Seoul National University, Suwon, Republic of Korea

**Keywords:** transducer of ErbB-2, gastric cancer, cell migration, invasion, Smad4, β-catenin

## Abstract

Transducer of ErbB-2.1 (Tob1), a tumor suppressor protein, is inactivated in a variety of cancers including stomach cancer. However, the role of Tob1 in gastric carcinogenesis remains elusive. The present study aimed to investigate whether Tob1 could inhibit gastric cancer progression *in vitro*, and to elucidate its underlying molecular mechanisms. We found differential expression of Tob1 in human gastric cancer (MKN28, AGS and MKN1) cells. The overexpression of Tob1 induced apoptosis in MKN28 and AGS cells, which was associated with sub-G1 arrest, activation of caspase-3, induction of Bax, inhibition of Bcl-2 and cleavage of poly (ADP-ribose) polymerase (PARP). In addition, Tob1 inhibited proliferation, migration and invasion, which were reversed in MKN1 and AGS cells transfected with Tob1 siRNA. Overexpression of Tob1 in MKN28 and AGS cells induced the expression of Smad4, leading to the increased expression and the promoter activity of p15, which was diminished by silencing of Tob1 using specific siRNA. Tob1 decreased the phosphorylation of Akt and glycogen synthase kinase-3β (GSK3β) in MKN28 and AGS cells, resulting in the reduced protein expression and the transcriptional activity of β-catenin, which in turn decreased the expression of cyclin D1, cyclin-dependent kinase-4 (CDK4), urokinase plasminogen activator receptor (uPAR) and peroxisome proliferator and activator receptor-δ (PPARδ). Conversely, silencing of Tob1 induced the phosphorylation of Akt and GSK-3β, and increased the expression of β-catenin and its target genes. Collectively, our study demonstrates that the overexpression of Tob1 inhibits gastric cancer progression by activating Smad4- and inhibiting β-catenin-mediated signaling pathways.

## Introduction

Tumor suppressor proteins are negative regulators of cell growth and can inhibit neoplastic transformation of cells. Unfortunately, most of the genes encoding various tumor suppressor proteins are inactivated during the course of carcinogenesis (1). Transducer of ErbB-2.1 (Tob1), is a tumor suppressor protein encoded by the *Tob* gene located on chromosome 17q21 (2). Tob1 is a member of the Tob/B cell translocation gene (BTG) family proteins, which upon overexpression arrests cell cycle in G1 phase (3–5). Tob1 binds to ErbB2 receptor and functions as a negative regulator of cell growth by blocking ErbB2-mediated cell signaling pathways (2). Yoshida *et al*(6) reported that mice lacking *Tob* gene developed spontaneous tumors primarily in the lung, liver and lymph nodes. Subsequent studies demonstrated that the expression of Tob1 is lost in various cancers including those of the breast (7), pancreas (8), thyroid (9) and stomach (10). While the loss of Tob1 has been implicated in the progression of human papillary thyroid carcinomas (9), ectopic expression of Tob1 inhibited cell cycle progression and reduced the growth of human breast cancer cell xenografts in nude mice (7). The anti-proliferative effect of Tob1 has been ascribed to its ability to inhibit the promoter activity of cyclin D1 (11).

The tumor suppressor function of Tob1 has largely been corroborated to its anti-proliferative effect on various cancer cells. However, the role of Tob1 in the migration and invasion of gastric cancer cells and its underlying mechanisms are yet to be investigated. Since Tob1 expression has been shown to be lost in human gastric carcinomas (10), we examined whether the restoration of Tob1 could prevent gastric cancer progression, and to elucidate the molecular mechanisms associated with its tumor suppressor function. It has been reported that Tob1 acts as a transcriptional co-activator by binding with Smad4 (11) and as a transcriptional co-repressor by interacting with β-catenin (12). Since functional inactivation of Smad4 (13–14) and aberrant expression of β-catenin (15) play pivotal roles in gastric carcinogenesis, we examined whether Tob1 could modulate the expression of Smad4 and β-catenin, thereby preventing gastric cancer progression. In the present study, we report that overexpression of Tob1 induces apoptosis and inhibits the proliferation, migration and invasion of gastric cancer cells, at least in part, by activating the expression of Smad4 and its target gene p15 as well as by downregulating the expression of β-catenin and its target genes, such as cyclin D1, uPAR and PPARδ.

## Materials and methods

### Cell culture and transfection

Human gastric cancer cell lines (AGS, MKN1 and MKN28) were purchased from the Korean Cell Line Bank (Seoul, Korea) and were maintained in RPMI-1640 medium supplemented with 10% fetal bovine serum at 37°C in 5% CO_2_. Cells were routinely checked for mycoplasma contamination. MKN28 and AGS cells were transiently transfected with myc-*Tob1* plasmid (2 or 4 *μ*g), which was a generous gift from Professor Jun-Mo Yang, Sungkyunkwan University, Seoul, Korea, using Genefectine™ reagent (Genetrone Biotech, Seoul, Korea) following the manufacturer’s instructions. Silencing of Tob1 in MKN1 and AGS cells was performed by transiently transfecting cells with 25 or 50 nM Tob1 si-RNA (Santa Cruz Biotechnology, Santa Cruz, CA, USA) by using DharmaFECT (Dharmacon, Lafayette, CO, USA) according to the manufacturer’s instructions.

### Western blot analysis

Cells were harvested and lysed with RIPA buffer [150 mM NaCl, 10 mM Tris (pH 7.2), 0.1% sodium dodecyl sulphate (SDS), 1% Triton X-100, 1% deoxycholate and 5 mM ethylenediaminetetraacetic acid (EDTA)] enriched with a complete protease inhibitor cocktail tablet (Roche Diagnostics, Mannheim, Germany), and then incubated on ice for 30 min with regular vortexing before centrifuging at 14,000 rpm at 4°C for 15 min. Protein concentration was determined by using bichinconinic acid (BCA) protein assay kit (Pierce Biotechnology, Rockford, IL, USA). The protein samples were boiled in 1X SDS sample buffer for 5 min for complete denaturation and were resolved on a 10 to 15% SDS-polyacrylamide gel according to the protocol described earlier (16). After electrophoresis, proteins were transferred onto polyvinyl difluoride (PVDF) membrane, which was blocked with 5% nonfat dry milk in 1X TBST (Tris-buffered saline with 0.1% Tween-20) and incubated with primary antibody at the appropriate final concentration followed by hybridization with horseradish peroxidase-conjugated anti-rabbit or anti-mouse secondary antibodies (1:5,000). Finally, western blot images were developed on photographic film using enhanced chemiluminescence (ECL) reagents. For each step, the membrane was washed with 1X TBST three times for 10 min. The primary antibodies used were: β-actin, β-catenin, Smad4, p27, CDK4, uPAR, Bax and Bcl-2 (Santa Cruz Biotechnology), Tob1 (Clone-4B1, Sigma Aldrich, St. Louis, MO, USA), p15, PPARδ (Abcam, Cambridge, UK), and Akt, pAkt, GSK3β, p-GSK3β (Ser-9), cleaved-polyadiporibosyl polymerase (PARP) and cyclin D1 (Cell Signaling Inc., Beverly, MA, USA).

### Total-RNA preparation and qRT-PCR

Total-RNA was extracted using TRIzol (Invitrogen, Carlsbad, CA, USA) and reversely transcribed to cDNA using the Superscript™ II First-Strand Synthesis System (Invitrogen). Following cDNA synthesis, qRT-PCR was performed as described (17) in a dual system LightCycler (Roche Applied Science, Mannheim, Germany) using the primers for *p15*, *Tob1*, *cyclin D1*, *uPAR* and *PPARδ. HPRT* was used for normalizing gene expression. All PCR primers and probe sequences (Universal Probe Library, Roche Applied Science) including the *HPRT* TaqMan probe (TIB Molbiol, Berlin, Germany) are listed in Table I.

### Cell proliferation assay

The effect of Tob1 overexpression on cell proliferation was measured by the water soluble tetrazolium salts (WST) method (EZ-Cytox kit; Daeil Lab Service, Seoul, Korea). MKN28 and AGS cells (1×10^5^), transfected with either myc-*Tob1* (2 or 4 *μ*g) or vector alone, were incubated in triplicate in a 12-well plate for 24 h at 37°C. MKN1 and AGS cells (1×10^5^) were transfected with either control si-RNA or Tob1 si-RNA (25 or 50 nM) in triplicate in a 12-well plate for 48 h at 37°C. EZ-Cytox solution (200 *μ*l) was added to each well and incubated for 80 min. The number of viable cells was measured in a 96-well plate at an optical density of 492 nm on a Sunrise reader (Tecan Trading AG, Mannedorf, Switzerland). Cell viability was described as the percentage of empty vector- or control si-RNA-transfected cells.

### Cell migration and invasion assay

Cells (5×10^4^) transfected with empty vector, myc-*Tob1* plasmid (2 or 4 *μ*g), *Tob1*si-RNA (25 or 50 nM) or control si-RNA, were subjected to Millipore’s (Billerica, MA, USA) 24-well Chemicon QCM™ cell migration assay and QCM™ fluorimetric cell invasion assay systems. After incubation at 37°C for 24 h, cell number was detected with a GENios Pro microplate reader (Tecan Trading AG) using 485/535 nm filter set. All migration and invasion assays were performed in triplicate in at least three independent experiments. Values are expressed as percentages of control (18).

### Wound healing assay

*In vitro* wound healing assay was performed to examine the migration of MKN28 and AGS cells transfected with either a control vector or myc-*Tob1*. In another experiment, AGS cells were transfected with either control si-RNA or Tob1 si-RNA. Transfected cells were grown on 6-well plates with their respective culture media. After the growing cell layers had reached confluence, wounds were prepared by a single scratch on the monolayer using a yellow pipette tip and washed the wounded layers with PBS to remove cell debris. We measured the closure or filling of the wounds at 0, 12 or 24 h using an Olympus IX71 fluorescence microscope with a TH4-200 camera. All experiments were performed in triplicate.

### Luciferase reporter gene assay

Cells were seeded into 12-well plates at a density of 1×10^5^ cells per well prior to transfection. Cells were transfected with TOPflash or FOPflash plasmid (kindly provided by Professor Sung-Hee Baek, College of Natural Sciences, Seoul National University, Seoul, Korea) or p15 promoter (a kind gift from Dr Joan Massague, Howard Hughes Medical Institute, Memorial Sloan-Kettering Cancer Center, NY, USA) together with an empty vector or myc-*Tob1* using Genefectin transfection reagent. pRL-TK (Promega, Madison, WI, USA) was used as a normalization control. After a further 24-h culture, the luciferase activity was measured using the Dual-Luciferase^®^ Reporter Assay System (Promega). For si-RNA experiments, cells were seeded into 12-well plates at a density of 1×10^5^ cells per well prior to transfection with either control-siRNA or Tob1-siRNA for 24 h followed by transfection with TOPflash or FOPflash plasmid (AGS cells) or p15 promoter construct (MKN1 and AGS cells) together with an normalization control pRL-TK for additional 24 h using Genefectin transfection reagent. The luciferase activity was measured by using the Dual-Luciferase Reporter Assay System according to the manufacturer’s instructions (Promega).

### The caspase-3 activity assay

The activity of caspase-3 in *Tob1*-transfected MKN28 and AGS cells was detected using Caspase-3 Colorimetric Activity Assay Kit (Millipore). The assay was performed in 96-well plates by incubating cell lysates (50 *μ*g) in 100 *μ*l reaction buffer containing caspase-3 substrate Ac-DEVD-pNA at 37°C for 2 h 30 min. The amount of p-nitroanilide, released by caspase activation, was quantified by measuring the optical density at 405 nm with a GENios Pro microplate reader (Tecan Trading AG). Lysis buffer: 20 mM HEPES, pH 7.5, 150 mM NaCl, 10% glycerol, 0.5% Nonidet P-40, 1 mM EDTA, 10 mM sodium fluoride, 10 mM β-glycerophosphate, 0.5 mM sodium orthovanadate, and 0.1 mM phenylmethylsulfonyl fluoride.

### Flow cytometry

In *Tob1*-transfected cells apoptosis was evaluated by flow cytometry. MKN28 or AGS (2×10^5^) cells were transfected with myc-*Tob1* plasmid (2 or 4 *μ*g) and cultured for 24 h. After 24 h, cells were harvested and fixed in cold 90% ethanol overnight at −20°C, and resuspended in staining buffer consisting of 100 mg/ml of RNase A (Quiagen, Valencia, CA, USA), 20 *μ*g/ml of propidium iodide (BD-Biosciences, San Jose, CA, USA), and 0.1% Nonidet P-40. The DNA content was analyzed by flow cytometry (BD-Biosciences).

### Annexin V staining

Annexin V staining was performed using FITC-Annexin V staining kit (BD-Biosciences) following the manufacturer’s instructions. Briefly, myc-*Tob1*-transfected cells were washed with PBS and resuspended in binding buffer containing Annexin V and propidium iodide. Flourescence intensity was measured using flow cytometry (BD-Biosciences).

### Statistical analysis

When necessary, data were expressed as mean ± SD of at least three independent experiments, and statistical analysis for single comparison was performed using the Student’s t-test. The criterion for statistical significance was ^*^p<0.05, ^**^p<0.01 and ^***^p<0.001, respectively, compared to corresponding control vector or control si-RNA.

## Results

### Tob1 inhibits proliferation of gastric cancer cells

**Figure 1:**
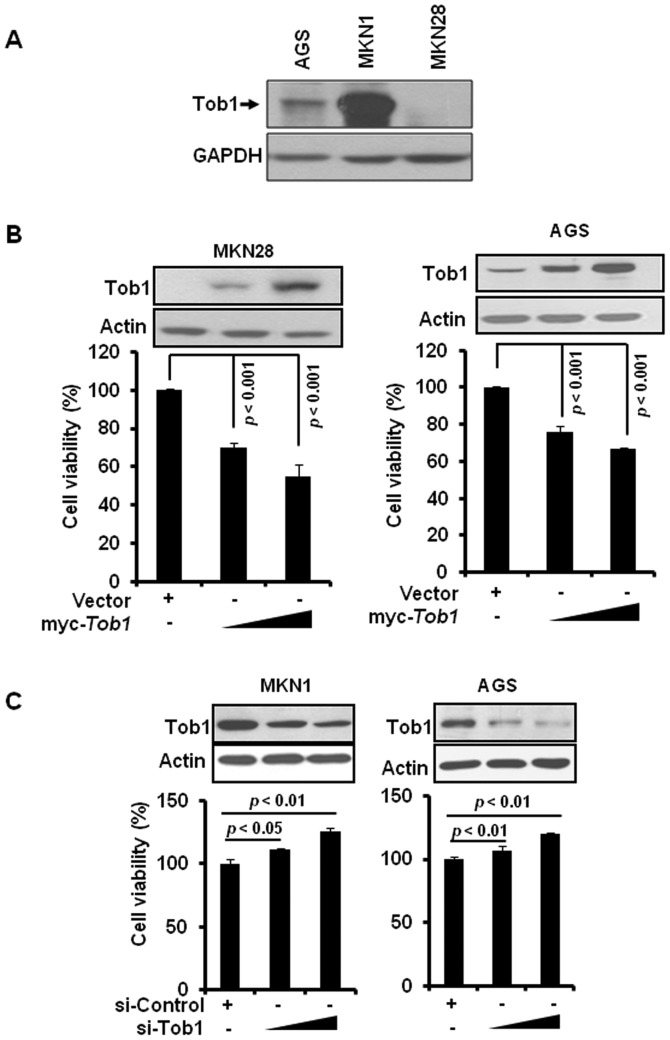
Tob1 inhibits proliferation of gastric cancer cells. (A) Protein lysates (30 *μ*g) were separated by SDS-PAGE and immunoblotted to detect the expression of Tob1 in AGS, MKN1 and MKN28 gastric cancer cells. (B) MKN28 or AGS cells were transiently transfected with myc-*Tob1* (2 or 4 *μ*g) or empty vector (4 *μ*g) for 24 h, and cell viability was evaluated using WST assay as described in the Materials and methods section. Western blot analysis (top panel) confirmed the expression of myc-*Tob1*. Data are representative of three independent experiments showing a similar pattern. (C) MKN1 and AGS cells were transfected with either control si-RNA (50 nM) or Tob1 siRNA (si-Tob1, 25 or 50 nM). Cell viability was assessed after 48 h of transfection. Western blot analysis (top panel) confirmed the downregulation of Tob1.

We first examined the expression of Tob1 in a variety of gastric cancer cells including MKN28, AGS, and MKN1. Tob1 was highly expressed in MKN1 cells and moderately expressed in AGS cells, while it was not detectable in MKN28 cells (Fig. 1A). To examine the role of Tob1 in gastric cancer, MKN28 and AGS cells were transiently transfected with myc-*Tob1*. Tob1 significantly decreased cell viability after 24 h in both MKN28 and AGS cells compared to vector alone (Fig. 1B). Conversely, the downregulation of Tob1 using specific si-RNA significantly increased the viability of MKN1 and AGS cells (Fig. 1C).

### Restoration of Tob1 induces apoptosis in gastric cancer cells

**Figure 2:**
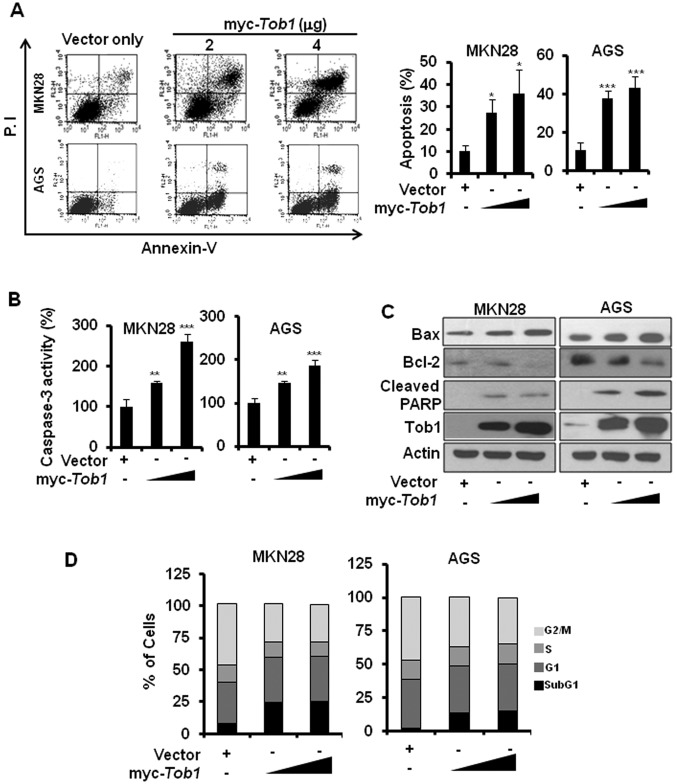
Tob1 induces apoptosis in gastric cancer cells. (A) Cells were transfected as mentioned above and were subjected to flow cytometric analysis upon staining with Annexin V and propidium iodide (PI). Right panel shows statistical analysis of apoptosis in cells transfected with control vector or myc-*Tob1*. ^*^p<0.05 and ^***^p<0.001, compared to respective control vector. (B) The caspase-3 activity was assessed from MKN28 and AGS cells transfected with myc-*Tob1* as indicated above. ^**^p<0.01 and ^***^p<0.001, compared to control vector. (C) The expression of Bax, Bcl-2, and cleavage of PARP after transfection of empty vector or myc-*Tob1* was investigated using their specific antibodies. Actin was used as loading control. (D) MKN28 and AGS cells transfected with empty vector or myc-*Tob1* were subjected to flow cytometry to analyze cell cycle distribution.

The inhibition of cell viability by ectopic expression of Tob1 prompted us to examine if Tob1 overexpression can induce apoptosis in gastric cancer cells. Annexin V staining revealed that Tob1 significantly induced apoptosis in both MKN28 and AGS cells (Fig. 2A). The caspase-3 activity assay using colorimetric substrate confirmed that Tob1 significantly induced apoptosis (Fig. 2B). In addition, Tob1 showed increased expression of Bax, reduced expression of Bcl-2 and induction of PARP cleavage (Fig. 2C). Furthermore, cell cycle analysis revealed the increased accumulation of *Tob1*-transfected MKN28 and AGS cells at the sub-G1 phase (Fig. 2D).

### Tob1 suppresses the migration and invasion of gastric cancer cells

**Figure 3:**
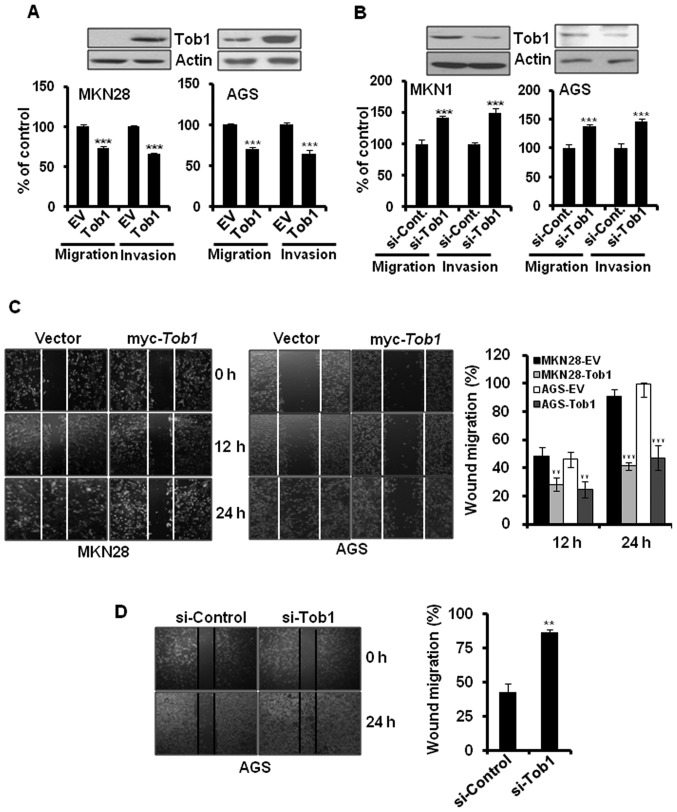
Tob1 inhibits migration and invasion of gastric cancer cells. (A) MKN28 and AGS cells were transfected with either myc-*Tob1* (4 *μ*g) or control vector (4 *μ*g) for 24 h. Cell migration and invasion assay was repeated three times as described in the Materials and methods. ^***^p<0.001 compared to respective empty vector (EV). Western blot analysis (top panel) confirmed the expression of myc-*Tob1*. (B) MKN1 and AGS cells were transiently transfected with either Tob1 si-RNA (50 nM) or control si-RNA (50 nM) for 48 h, and cell migration and invasion assay was performed in triplicate as described in the Materials and methods. ^**^p<0.01 and ^***^p<0.001 compared to respective si-control. Western blot analysis (top panel) confirmed downregulation of Tob1. (C) MKN28 or AGS cells were transfected with myc-*Tob1* (4 *μ*g) or empty vector (4 *μ*g) for 12 and 24 h, respectively. Wound healing assay was performed as described in the Materials and methods. ^**^p<0.01 and ^***^p<0.001 compared to empty vector (EV) at the indicated time. (D) AGS cells were transfected with control si-RNA (50 nM) or Tob1 si-RNA (50 nM) for 24 h and scraped for wound healing assay as described in the Materials and methods section. ^**^p<0.01 compared to control-siRNA.

To evaluate the role of Tob1 in gastric cancer progression, myc-*Tob1*-transfected MKN28 and AGS cells were subjected to migration and invasion assay. Overexpression of Tob1 significantly inhibited the migration and invasion of MKN28 and AGS cells (Fig. 3A). In addition, knock-down of Tob1 using specific si-RNA increased the migration and invasion of MKN1 and AGS cells by about 40% (Fig. 3B). These findings were further confirmed by the wound healing assay. The overexpression of Tob1 significantly inhibited the migration of MKN28 and AGS cells at 24 h after transfection (Fig. 3C). In addition, we examined the knock-down effect of Tob1 in AGS cells alone because MKN28 cells did not express endogenous Tob1. The downregulation of Tob1 using specific si-RNA increased cell migration compared to control si-RNA (Fig. 3D).

### Overexpression of Tob1 increases the expression of Smad4 and its target gene the p15

**Figure 4:**
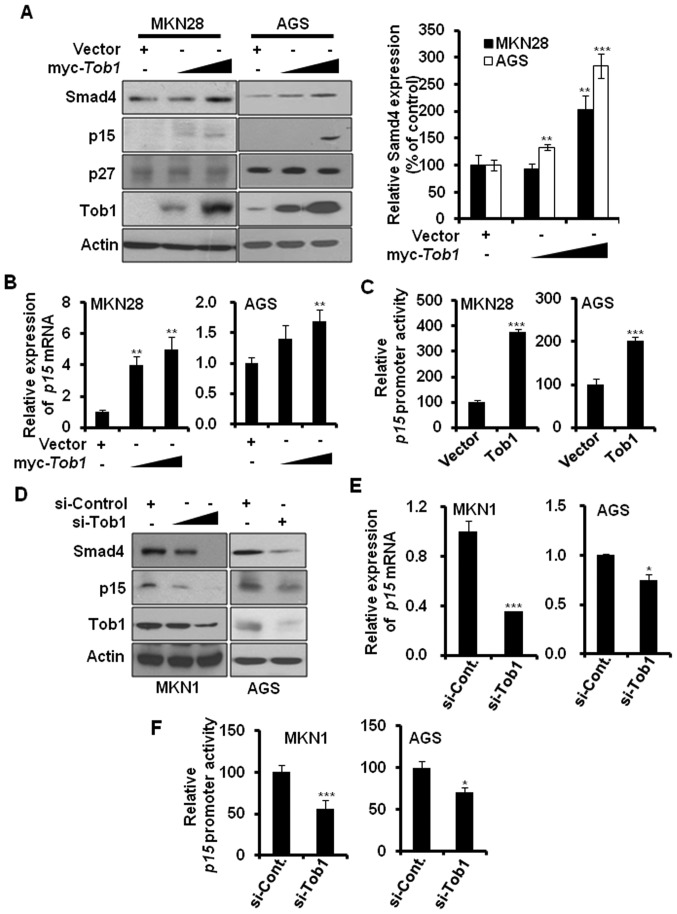
Tob1 enhances the expression of Smad4 and p15 in gastric cancer cells. (A) Protein lysates (30 *μ*g) from MKN28 or AGS cells transfected with either control vector or myc-*Tob1* (2 or 4 *μ*g) were separated by 10–15% SDS-PAGE, and the expression of Smad4 and its target gene products, p15 and p27, was detected by immunoblot analysis. Data are representative of three independent experiments. ^**^p<0.01 and ^***^p<0.001 compared to respective control vector. (B) Total-RNA was isolated from MKN28 or AGS cells transfected with either vector alone or myc-*Tob1* (2 or 4 *μ*g) and qRT-PCR was performed to assess the expression of *p15* mRNA. *HPRT* was used for normalizing gene expression. ^**^p<0.01, compared to control vector. (C) The luciferase activity assay using p15 promoter was performed in the presence or absence of myc-*Tob1* (0.4 *μ*g). ^***^p<0.001, compared to control vector. (D) MKN1 and AGS cells were transiently transfected with either control si-RNA (50 nM) or Tob1 si-RNA (25 and 50 nM for MKN1, 50 nM for AGS) for 48 h, and cell lysates were separated by SDS-PAGE and immunoblotted using their specific antibodies. Data are representative of three independent experiments. (E) Total-RNA was isolated from MKN1 or AGS cells transfected with either control si-RNA (50 nM) or Tob1 si-RNA, and qRT-PCR was performed to assess the expression of *p15* mRNA. *HPRT* was used as internal control. ^*^p<0.05 and ^***^p<0.001, as compared to respective control si-RNA. (F) The luciferase activity assay using p15 promoter was performed after transfection of control si-RNA (50 nM) or Tob1 si-RNA (50 nM) for 48 h. The p15 promoter activity assay was performed in triplicate. ^*^p<0.05 and ^***^p<0.001, compared to control si-RNA.

It has been reported that Tob1 interacts with Smad4 (11), a tumor suppressor protein that is inactivated during gastric cancer progression (14). Therefore, we investigated whether Tob1 could regulate the expression of Smad4. Ectopic expression of Tob1 significantly elevated the expression of Smad4 in a dose-dependent manner in both MKN28 and AGS cells (Fig. 4A), but did not alter Smad4 mRNA expression (data not shown). In addition, we examined the effect of Tob1 on the expression of Smad4 target genes. As shown in Fig. 4A, transient overexpression of Tob1 increased the expression of p15, but not p27. qRT-PCR analysis showed that the expression of p15 was also increased at transcriptional level in both MKN28 and AGS cells (Fig. 4B). The luciferase activity assay using p15 promoter further confirmed that Tob1 significantly increased the p15 promoter activity (Fig. 4C). The role of Tob1 in the regulation of the expression of Smad4 and its target gene p15 was further confirmed by transfecting MKN1 and AGS cells with Tob1 siRNA. The knock-down of Tob1 significantly reduced the expression of Smad4 and p15 in both MKN1 and AGS cells as compared to cells harboring control si-RNA (Fig. 4D). In addition, in comparison to control si-RNA, the relative expression of p15 mRNA (Fig. 4E) and the p15 promoter activity (Fig. 4F) were significantly decreased in both MKN1 and AGS cells transfected with Tob1 siRNA.

### Tob1 inhibits β-catenin-mediated signaling in gastric cancer cells

**Figure 5:**
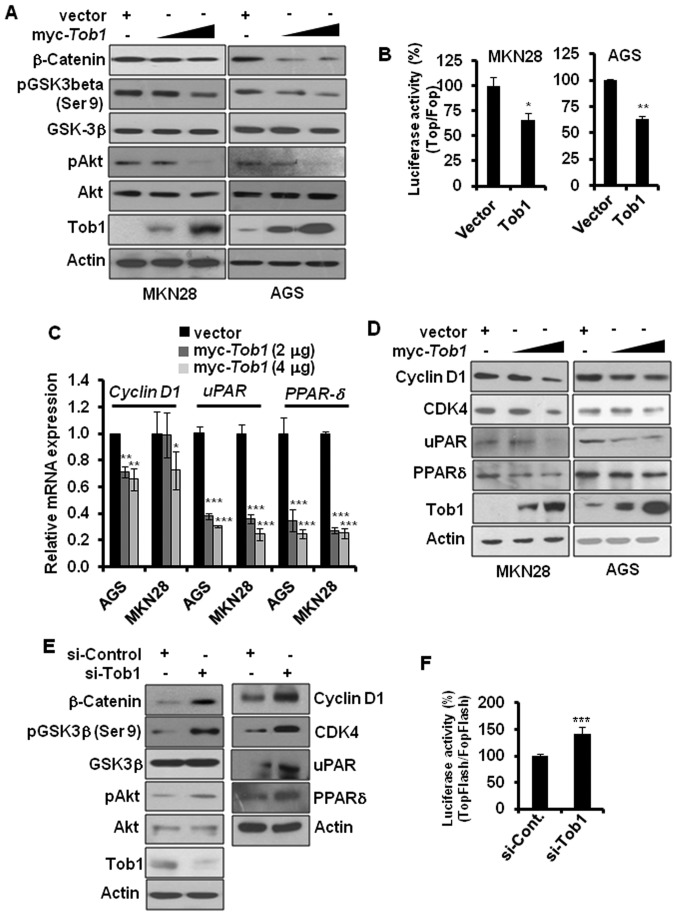
Tob1 inhibits β-catenin signaling in gastric cancer cells. (A) Protein lysates (15 *μ*g) from MKN28 or AGS cells transfected with myc-*Tob1* (2 or 4 *μ*g) were separated by 10% SDS-PAGE, and the expression of β-catenin, pAkt and pGSK3β-(serine-9) was detected using their specific antibodies. Data are representative of three different experiments. (B) Cells (MKN28 and AGS) were transfected with luciferase constructs harboring TopFlash or FopFlash along with control vector or myc-*Tob1* (0.4 *μ*g). The luciferase activity assay was performed as described in the Materials and methods. ^*^p<0.05 and ^**^p<0.01 compared to respective control vector. (C) Total-RNA was isolated from MKN28 or AGS cells transfected with either vector alone or myc-*Tob1* (2 or 4 *μ*g) and qRT-PCR was performed to assess the mRNA expression of *cyclin D1*, *uPAR* and *PPARδ*. Gene expression was normalized to the level of *HPRT*. ^*^p<0.05, ^**^p<0.01 and ^***^p<0.001 compared to control vector. (D) Protein lysates (30 *μ*g) from MKN28 and AGS cells transfected with either control vector or myc-*Tob1* (2 or 4 *μ*g) were subjected to western blot analysis to detect the expression of cyclin D1, CDK4, uPAR and PPARδ. Data are representative of three different experiments. (E) AGS cells were transiently transfected with either control si-RNA or Tob1 si-RNA for 48 h, and cell lysates were separated by 10% SDS-PAGE to determine the expression of β-catenin, pAkt, pGSK-3β-(serine-9), cyclin D1, CDK4, uPAR and PPARδ. Data are representative of three independent experiments. (F) AGS cells were transfected with luciferase constructs harboring TopFlash or FopFlash with control si-RNA (25 nM) or Tob1 si-RNA (25 nM). The luciferase activity assay was performed as described in the Materials and methods. ^*^p<0.001 (vs. control si-RNA).

Aberrant activation of β-catenin-mediated signaling has been implicated in gastric cancer progression (15). Overexpression of Smad4 has been reported to diminish β-catenin signaling, and, hence, prevent tumor progression (19). Since restoration of Tob1 elevated the expression of Smad4, we investigated the role of Tob1 in the regulation of β-catenin-mediated signaling. Overexpression of Tob1 decreased the expression of β-catenin in MKN28 and AGS cells (Fig. 5A). β-catenin is degraded by GSK3β, an enzyme inactivated via phosphorylation of its serine-9 residue by upstream kinase Akt (20). We examined whether the decreased expression of β-catenin in *Tob1*-overexpressing gastric cancer cells could result from the reduced phosphorylation of Akt and GSK3β. As expected, Tob1 decreased the phosphorylation of Akt and GSK3β (Fig. 5A). Ectopic expression of Tob1 not only reduced the expression of β-catenin but also attenuated β-catenin-mediated transcriptional activity. The luciferase activity assay using TopFlash or FopFlash along with control vector or myc-*Tob1* showed that Tob1 significantly reduced the transcriptional activity of β-catenin/T cell factor (TCF) complex (Fig. 5B), which resulted in decreased expression of β-catenin target genes, such as *cyclin D1*, *uPAR* and *PPARδ* in both qRT-PCR and western blot analyses (Fig. 5C and D). The knock-down of Tob1 using specific si-RNA increased the phosphorylation of Akt and GSK3β (serine-9), resulting in the increased expression of β-catenin and its target gene in AGS cells (Fig. 5E). In addition, the luciferase activity assay using TopFlash vector after knock-down of Tob1 using specific si-RNA in AGS cells significantly increased the transcriptional activity of β-catenin (Fig. 5F), verifying again that Tob1 negatively regulates β-catenin signaling pathway.

## Discussion

Gastric cancer is the second leading cause of cancer-related deaths in the world (21). Inactivation of tumor-suppressor genes is one of the critical events in the development and progression of gastric cancer (14). Thus, restoration of tumor suppressor gene function is considered as a rational approach for therapeutic intervention of carcinogenesis. For instance, the pharmacological activation of a tumor suppressor protein, p53, is being widely studied for the development of new cancer chemotherapeutics (22). Like many other tumor suppressor proteins, the expression of Tob1 is frequently lost in various cancers. A recent study by Yu *et al*(10) demonstrated that Tob1 is either absent or expressed at reduced level in 75% of primary gastric cancer cases. However, the role of Tob1 in gastric cancer progression and its underlying mechanisms have not been fully clarified. Thus, we attempted to investigate whether ectopic expression of Tob1 could inhibit gastric cancer progression and to elucidate its underlying mechanisms. Here, we report for the first time that ectopic expression of Tob1 induces apoptosis and inhibits the proliferation of gastric cancer cells *in vitro*. Our results showed that Tob1 induced Bax expression and inhibited Bcl-2 expression in gastric cancer cells. Similar effects of Tob1 on Bax and Bcl-2 expression in human breast cancer cells have been reported earlier (23). In addition, we found a novel mechanism showing that Tob1 induced PARP cleavage by promoting caspase-3 activity, leading to the apoptosis of gastric cancer cells. The increased migration and invasion of cancer cells has been known to be directly associated with poor prognosis. Thus, the restoration of Tob1 in gastric cancer patients could delay the disease progression and improve the prognosis by inhibiting migration and invasion of cancer cells.

The expression of Smad4 has been known to be reduced in gastric cancer (14), and re-expression of Smad4 has been reported to inhibit tumor progression (24–25). In this study, we found that Tob1 induced the expression of Smad4, which increased the expression of p15, a CDK inhibitor, at transcriptional level. Since Tob1 has been known to increase the Smad4-mediated transcriptional activity by interacting with Smad4 (11), the induction of p15 expression by Tob1 may be due to the enhancement of transcriptional activity of Tob1-Smad4 complex. Thus, the induction of Smad4 expression by Tob1 in MKN28 and AGS cells could inhibit gastric cancer progression through activation of Smad4-mediated signaling. In addition, Smad4 has been shown to inhibit the expression of β-catenin (19), which is implicated in gastric cancer progression (15). The downregulation of β-catenin expression by Tob1 suggests that Tob1 can interfere with β-catenin-mediated signal transduction pathways. Our study revealed that Tob1 decreased the expression of β-catenin-target genes including cyclin D1, CDK4, uPAR, and PPARδ. While cyclin D1 and CDK4 are well known biomarker of cell proliferation, uPAR (26) and PPARδ have been reported to play role in the migration and invasion of gastric cancer cells (27). Thus, the anti-proliferative effect of Tob1 in gastric cancer might be due to the decreased expression of cyclin D1 and CDK4, and the increased expression of p15, while the anti-migratory and anti-invasive potential of Tob1 may be mediated through the inhibition of uPAR and PPARδ in gastric cancer cells. Besides uPAR, which degrades extracellular matrix and facilitates gastric cancer progression (28,29), matrix metalloproteinases (MMPs) have also been implicated in the migration and invasion of gastric cancer (30). However, the expression and the activity of MMP-2/-9 in gastric cancer cells remained unaffected by Tob1 overexpression (data not shown).

A recent study demonstrated that overexpression of Tob1 in lung cancer cells increased the expression of another tumor suppressor protein phosphatase and tensin homolog (PTEN) (31), which is a negative regulator of Akt. In addition, re-expression of PTEN in prostate cancer (PC3) cells downregulated β-catenin expression (32). Thus, it would be worthwhile to investigate whether the decreased phosphorylation of Akt and the reduced expression of β-catenin upon overexpression of Tob1 in gastric cancer cells also results from the induction of PTEN.

**Figure 6:**
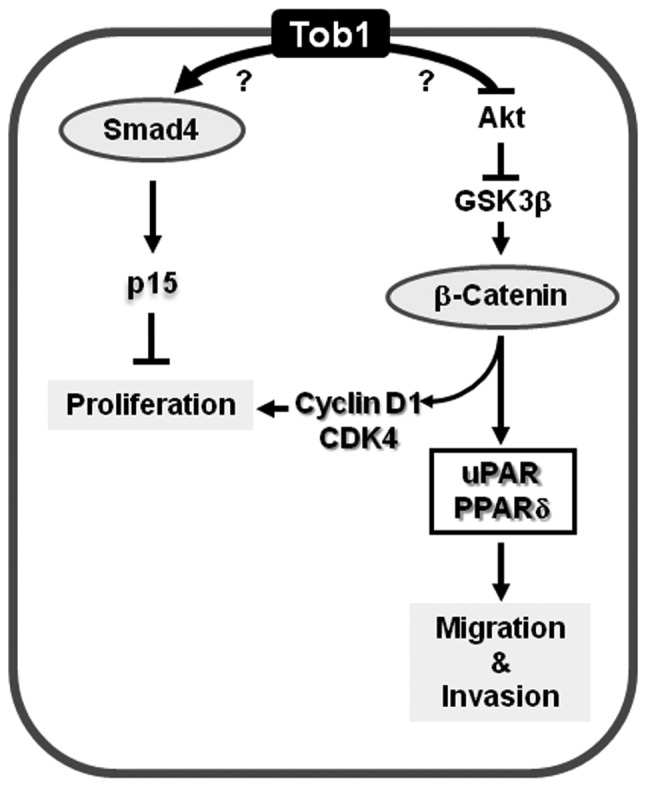
A schematic model showing the role of Tob1 in the modulation of Smad4- and β-catenin-mediated signal transduction pathways and its impact on apoptosis, proliferation, migration and invasion of gastric cancer cells.

In conclusion, the present study demonstrates that Tob1 functions as a tumor suppressor protein in gastric cancer cells, at least in part, by inducing apoptosis and inhibiting proliferation, migration and invasion via the activation of Smad4- and suppression of β-catenin-mediated signaling pathways (Fig. 6).

## Figures and Tables

**Table I:** Primer sequences for quantitative RT PCR.

Gene	Orientation	Sequences	Tm (°C)
*Tob1*	Forward	5′-TCTGTATGGGCTTGGCTTG-3′	54.9
	Reverse	5′-TGTTGCTGCTGTGGTGGT-3′	54.3
*CDKN2B*	Forward	5′-CAACGGAGTCAACCGTTTC-3′	54.9
	Reverse	5′-GGTGAGAGTGGCAGGGTCT-3′	54.2
*Cyclin D1*	Forward	5′-GAAGATCGTCGCCACCTG-3′	56.6
	Reverse	5′-GACCTCCTCCTCGCACTTCT-3′	59.5
*uPAR*	Forward	5′-ACACCACCAAATGCAACGA-3′	52.7
	Reverse	5′-CCCCTTGCAGCTGTAACAC-3′	57.1
*PPARδ*	Forward	5′-GGGAAAAGTTTTGGCAGGAG-3′	55.4
	Reverse	5′-TGCCCAAAACACTGTACAACA-3′	53.9
*HPRT*	Forward	5′-CTCAACTTTAACTGGAAAGAATGTC-3′	54.1
	Reverse	5′-TCCTTTTCACCAGCAAGCT-3′	55.5
